# A Novel GFDM Waveform Design Based on Cascaded WHT-LWT Transform for the Beyond 5G Wireless Communications

**DOI:** 10.3390/s21051831

**Published:** 2021-03-05

**Authors:** Meryem Maraş, Elif Nur Ayvaz, Meltem Gömeç, Asuman Savaşcıhabeş, Ali Özen

**Affiliations:** 1 Department of Electrical and Electronics Engineering, Nuh Naci Yazgan University—HARGEM, Kayseri 38010, Turkey; 20101023@ogrenci.nny.edu.tr (M.M.); 20101022@ogrenci.nny.edu.tr (E.N.A.); ahabes@nny.edu.tr (A.S.); 2 Tasarruf A. Ş., Gevhernesibe, İstasyon Cd. No:41/C, Kayseri 38010, Turkey; meltem.gomec@tasarruf.com.tr

**Keywords:** waveform design, beyond 5G, GFDM, lifting wavelet transform, WHT-GFDM, OFDM

## Abstract

In this paper, a new WHT-LWT-GFDM waveform obtained by combining Walsh–Hadamard Transform (WHT), Lifting Wavelet Transform (LWT), and Generalized Frequency Division Multiplexing (GFDM) is presented for use in next-generation wireless communication systems. The proposed approach meets the requirement of 5th-generation (5G) and beyond communication schemes in terms of low latency, low peak-to-average-power ratio (PAPR), and low bit-error rate (BER). To verify the performance of the presented waveform, PAPR and BER simulation results were obtained in additive white Gaussian noise (AWGN) and flat Rayleigh fading channels, and the performance of the proposed system was compared with conventional Orthogonal Frequency Division Multiplexing (OFDM), GFDM, and Walsh–Hadamard transform-based GFDM (WHT-GFDM). Simulation results show that the proposed waveform achieves the best BER and PAPR performances and it provides considerable performance gains over the conventional waveforms.

## 1. Introduction

Wireless communication systems face severe necessities in terms of transmission speed, technique capacity, reliability, and latency to meet the communication of new rising implementations such as Internet of Things (IoT), ultra-high description multimedia and virtual reality (VR) in the fifth generation (5G) [1,2,3]. Moreover, the development of wireless communication technique is limited to several harmful factors in the radio propagation environments, such as inter-symbol interference (ISI), multi-user interference (MUI), propagation attenuation, and multipath fading [4]. These damaging components cause various technological difficulties in the plan of reliable wireless communication systems. A heuristic approach to dealing with these design challenges is the use of a multi-antenna communication paradigm, which is of great interest in both academia and industry, because of obvious demonstration advantages of network capacity and service quality (QoS) [5]. The multi-input multi-output (MIMO) technique has been built by mounting multiple antennas to the transmitter and receiver terminals [6], which have proven to provide a range of improvements over single-input–single-output (SISO) systems [7].

However, Generalized Frequency Division Multiplexing (GFDM) waveform was recommended for the 5G air interface due to the nominal system complexity, suppleness spectrum coordination, and lower peak-to-average-power ratio (PAPR), which provides higher power efficiency compared to OFDM [8]. The flexibility of the GFDM simplifies association with the single-carrier frequency domain equalizer (SC-FDE) and the filter bank multi-carrier (FBMC) systems [9]. The GFDM is based on modulation per subcarrier, where every subcarrier is single and independently modulated with multiple symbols. Subsequently, in this modulation scheme, the subcarrier is filtered through a circularly shifted prototype filter in the time and frequency regions. This filtering is intended to reduce the out-of-band emission (OOBE) residues and thus facilitates split-spectrum and dynamic spectrum distributions without causing serious interference to the GFDM system or other users. On the other hand, both ISI and inter-carrier interference (ICI) between sub-carriers may occur with such a filtering process. Fortunately, to obtain better performance than OFDM in several applications, used interference reduction techniques such as matched filter receiver with repetitive interference reduction, which is establish be effective, can be exploited well-designed receiving techniques [10].

In the challenge between the 5G waveform applicants, various modulation techniques were proposed. Some of the waveforms, for example; the FBMC system, which allows for simultaneous multiple access, focuses on providing a very low OOBE [11]. Other designs, such as windowed-OFDM (W-OFDM) [12] and filtered-OFDM (F-OFDM) [13], deal with the application complexity leading to several OFDM-based recommendations. In addition, GFDM was proposed as an alternative to OFDM to increase spectral efficiency by decreasing the cyclic prefix (CP) of OFDM [14]. However, due to the flexibility in adjusting dissimilar parameters such as subcarrier and subscript number, prototype pulse shape, and active subscript set, GFDM can be reconfigured to meet dissimilar necessities [15]. This flexibility performs the design and implementation of the combined multi-carrier frame based on the GFDM model.

In this study, it is recommended to combine Walsh–Hadamard transform (WHT) [16] and Lifting Wavelet Transformation (LWT) [17] with GFDM waveform to increase the achievement of multi-carrier wireless telecommunication schemes using GFDM scheme. To examine the performance of the proposed WHT-LWT-GFDM waveform and to compare with waveforms such as conventional OFDM, GFDM and WHT-GFDM [18], using WHT, the computer simulation studies are performed over additive white Gaussian noise (AWGN) and frequency flat Rayleigh fading channels. It is shown that the acquired performances with the recommended waveform are highly satisfactory from the computer simulation outcomes.

The rest of our study is organized as follows: GFDM waveform construction is explicated in Section 2. Walsh–Hadamard Transform is summarized in Section 3. Lifting Wavelet Transform is described in Section 4. The construction of the proposed waveform is explained in detail in Section 5. Computer simulations are shown in Section 6. In the last section, the results of computer simulations are discussed comparatively.

## 2. Generalized Frequency Division Multiplexing (GFDM) Waveform Structure

**Figure 1:**
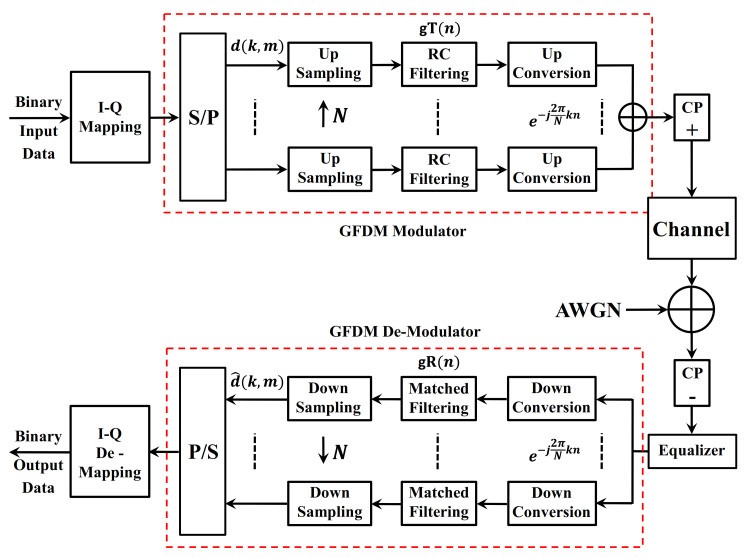
The block scheme of the GFDM waveform transceiver structure.

The main transceiver block scheme of the communication system using the GFDM waveform is shown in Figure 1.

Randomly generated serial input binary data on the transmitter side of the GFDM system given in Figure 1 are modulated by the I-Q mapping block. The modulated data is mapped in the GFDM modulator block, each subcarrier is shaped with Raised Cosine (RC) filter as a pulse shaping filter and the subcarrier is modulated with the center frequency of $e^{-j2\pi kn/N}$. To fulfill the Nyquist criteria, each symbol is sampled at the level N, which gives a vector of MN in the sublayer. The pulse shaping filter is selected to have M×N periodicity. Different pulse response filters can be used to shape sub-carriers, and this method affects OOBE and Symbol Error Rate (SER) performance. GFDM is based on the modulation of individual blocks, where each block consists of a series of sub-carriers and sub-symbols. The sub-carriers are filtered with RC filter as a pulse shaping filter that is shifted cyclically in the time and frequency region. This procedure allows for split-spectrum and dynamic spectrum allocation by reducing OOBE, without causing serious interference in embedded services or other users. After the data symbols are placed in a $d\left(k,m\right)$ column, all symbols are summed and the GFDM signal is generated. In the communication system, $d\left(k,m\right)$ contains complex data symbols, K carriers and M sub-symbols [8]. We obtain the matrix d concatenating these columns:

(1) $$d=\left[\begin{array}{ccc} d\left(0,0\right) & \cdots & d\left(0,M-1\right)\\ \vdots & \ddots & \vdots \\ d\left(K-1\right) & \ldots & d\left(K-1,M-1\right) \end{array}\right]$$

The K×M matrix will be treated as an information block, i.e., k=0…K−1 indexes the sub-carriers and m=0…M−1 indexes the elements of a time slot.

The transmission signal can be expressed as;

(2) $$x\left(n\right)=\overset{M-1}{\underset{m=0}{\sum }}\overset{K-1}{\underset{k=0}{\sum }}d\left(k,m\right)\left[gT\left(n-mN\right)\mathrm{mod}NM\right]e^{-j\frac{2\pi }{N}kn},\;0\leq n\leq NM-1$$

Through the frequency selective channel, the obtained GFDM signal is corrupted by AWGN. Assuming $y\left(n\right)$ are the time samples acquired at the receiver, data equalization is done at the receiver where $\hat{d}\left(k,m\right)$ are obtained by inverting the frequency shift, implementing the matched filter $gR\left(n\right)$ and down sampling. Thus, yielding signal at n=mN is defined as:

(3) $$\hat{d}\left(k,m\right)=\left(y\left(n\right)e^{-j\frac{2\pi }{N}kn}\right)⊛gR\left(n\right){|}_{n=MN}$$

where ⊛ denotes circular convolution with respect to n [19]. After correcting the corrupted data, the receiver performs the reverse operation to recover the signal as shown in Figure 1.

## 3. Walsh–Hadamard Transform

**Figure 2:**
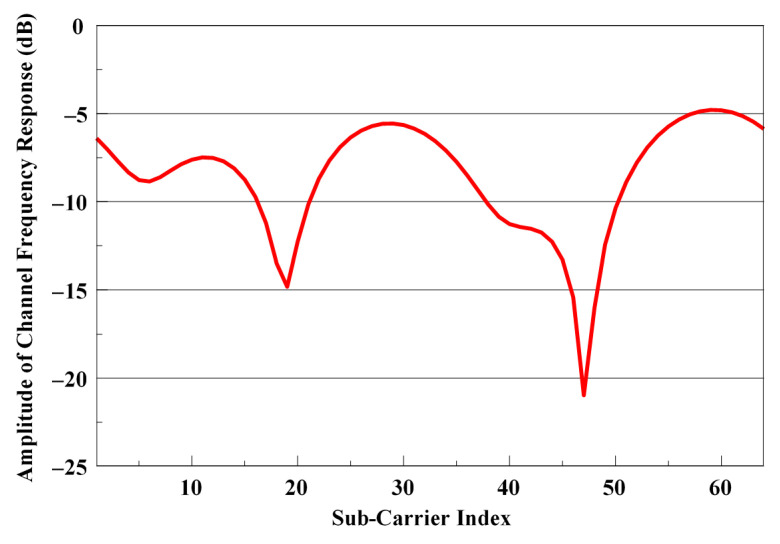
Frequency response of the wireless communication channel.

Due to the disruptive effects of the multipath propagation created by the wireless communication channel, very deep fading may cause distortion to the received signal. These deep fading is one of the biggest factors that reduce the system performance, especially in multi-carrier communication systems such as OFDM, due to the required high signal to noise ratio. As the frequency response of a wireless communication channel shown in Figure 2 shows that some sub-carriers become very weak at the receiver, these sub-carriers cannot be recovered and therefore due to this information loss, the system performance decreases. In such a channel, to improve performance, the missing information must be recovered in some way.

Without applying any correction or equalization, even if some sub-carriers are completely lost, what needs to be done is to load each bit of information to all sub-carriers at different rates to be sent to the receiver. Thus, even if some sub-carriers are completely lost, it will be possible to recover them as the information they carry is partially loaded on other carriers. One way to accomplish this is to use the Walsh–Hadamard Transform (WHT), which is an orthogonal transformation [20,21].

Although there are different orthogonal transformations, the Walsh–Hadamard Transform was used in this study. There are some important reasons for using WHT:

- It is an orthogonal transformation,
- The transformation matrix is a square matrix its dimensions are 2^A^ (A = 0, 1, 2, …),
- The transformation matrix can be recursively defined in any size,
- Since the matrix elements consist of 1 and −1, the transformation process can be performed only by addition and subtraction without the need for multiplication,
- Since there is no relationship between any two rows or any two columns of the transformation matrix, the information loaded on the sub-carriers is also different from each other,

Because WHT matrix is square matrix and is the size of the powers of 2, it is easy to apply to multi-carrier systems.

Walsh–Hadamard transformation matrix,

(4) $$W_{1\times 1}=\left[1\right]$$

including,

(5) $$W_{2\times 2}=\left[\begin{array}{c} W_{1\times 1}\;W_{1\times 1}\\ W_{1\times 1}-W_{1\times 1} \end{array}\right]=\left[\begin{array}{c} 1\;1\\ 1-1 \end{array}\right]$$

and

(6) $$W_{2K\times 2K}=\left[\begin{array}{c} W_{K}\;W_{K}\\ W_{K}-W_{K} \end{array}\right]$$

can be acquired in desired size as.

The Walsh–Hadamard Transform of a sequence $\left\{v\left[n\right]\right\}$, consisting of N samples, can be given by the Equation (7).

(7) $$V\left(k\right)=\overset{N-1}{\underset{n=0}{\sum }}v\left[n\right]W\left(n,k\right),\;k=0,\;1,\;\ldots ,\;N-1$$

where $W\left(n,k\right)$ describes the Walsh–Hadamard transform. Similarly, the inverse Walsh–Hadamard Transform can also be given with Equation (8).

(8) $$v\left[n\right]=\frac{1}{N}\overset{N-1}{\underset{k=0}{\sum }}V\left(k\right)W\left(n,k\right),\;n=0,\;1,\;\ldots ,\;N-1$$

Walsh–Hadamard Transform is applied in the frequency region in OFDM or multi-carrier communication system. The data to be sent is spread to all sub-carriers by WHT process before Inverse Fast Fourier Transform (IFFT) process. In the receiver, after Fast Fourier Transform (FFT) and channel equalization, the spread data over the sub-carriers will be recovered by the Inverse Walsh–Hadamard Transform (IWHT) process.

Data string to be sent in an OFDM signal or multi-carrier communication signal is defined as Equation (9).

(9) $$D=\left[D_{0}D_{1}\cdots D_{62}D_{63}\right]$$

Including the 64 × 64 size WHT matrix in (10),

(10) $$W=\left[\begin{array}{ccccc} w_{0,0} & w_{0,1} & \cdots & w_{0,62} & w_{0,63}\\ w_{1,0} & w_{1,1} & \cdots & w_{1,62} & w_{1,63}\\ \vdots & \vdots & \ddots & \vdots & \vdots \\ w_{62,0} & w_{62,1} & \cdots & w_{62,62} & w_{62,63}\\ w_{63,0} & w_{63,1} & \cdots & w_{63,62} & w_{63,63} \end{array}\right]$$

the array to be obtained at the end of the WHT process can be expressed as in (11).

(11) $$D^{\prime }=DW$$

Thus, the corresponding data is obtained as in (12).

(12) $${\left[\begin{array}{c} {D^{\prime }}_{0}\\ {D^{\prime }}_{1}\\ \vdots \\ {D^{\prime }}_{62}\\ {D^{\prime }}_{63} \end{array}\right]}^{T}={\left[\begin{array}{c} D_{0}\\ D_{1}\\ \vdots \\ D_{62}\\ D_{63} \end{array}\right]}^{T}\left[\begin{array}{ccccc} w_{0,0} & w_{0,1} & \cdots & w_{0,62} & w_{0,63}\\ w_{1,0} & w_{1,1} & \cdots & w_{1,62} & w_{1,63}\\ \vdots & \vdots & \ddots & \vdots & \vdots \\ w_{62,0} & w_{62,1} & \cdots & w_{62,62} & w_{62,63}\\ w_{63,0} & w_{63,1} & \cdots & w_{63,62} & w_{63,63} \end{array}\right]$$

Inside each element of this new array in (12) is a piece of all the elements of the first array. Therefore, even if some elements of the new array are completely lost due to deep fading in the channel, it may be possible to recover all elements of the old array.

In the receiver, if the data string at the channel equalizer output is represented by the vector ${\tilde{D}}^{\prime }$, then the data string at the IWHT output is obtained as:

(13) $$\hat{D}=\frac{1}{64}\tilde{D^{\prime }}W$$

To put it more clearly,

(14) $${\left[\begin{array}{c} {\hat{D}}_{0}\\ {\hat{D}}_{1}\\ \vdots \\ {\hat{D}}_{62}\\ {\hat{D}}_{63} \end{array}\right]}^{T}=\frac{1}{64}{\left[\begin{array}{c} {{\tilde{D}}^{\prime }}_{0}\\ {{\tilde{D}}^{\prime }}_{1}\\ \vdots \\ {{\tilde{D}}^{\prime }}_{62}\\ {{\tilde{D}}^{\prime }}_{63} \end{array}\right]}^{T}\left[\begin{array}{ccccc} w_{0,0} & w_{0,1} & \cdots & w_{0,62} & w_{0,63}\\ w_{1,0} & w_{1,1} & \cdots & w_{1,62} & w_{1,63}\\ \vdots & \vdots & \ddots & \vdots & \vdots \\ w_{62,0} & w_{62,1} & \cdots & w_{62,62} & w_{62,63}\\ w_{63,0} & w_{63,1} & \cdots & w_{63,62} & w_{63,63} \end{array}\right]$$

is obtained as.

## 4. Lifting Wavelet Transform

Though wavelet transform has been used in signal processing implementations for long time, it has begun to stand out in the area of communication in recent years. Because of the pliable construction of the wavelet transformation and the adaptive time-frequency intervals on the sub-carriers, the discrete wavelet transform (DWT)-based OFDM (DWT-OFDM) construction is an alternative to the FFT-based OFDM construction [22].

The DWT converts discrete data from time domain to time and frequency domain. On account of many interesting attributes, the discrete wavelet transformation is being used in many possible applications as for example voice compression, which gives quicker sending in mobile telecommunications. DWT is employed in medical applications for its real-time processing abilities. Besides the two applications already mentioned wavelets are used in other applications as well, as for example: in denoising, edge detection, echo cancellation, etc.

An alternative method for constructing biorthogonal wavelet transform, the lifting scheme [23,24], has some advantages over the classical standard wavelet transform which are given below.

**Figure 3:**
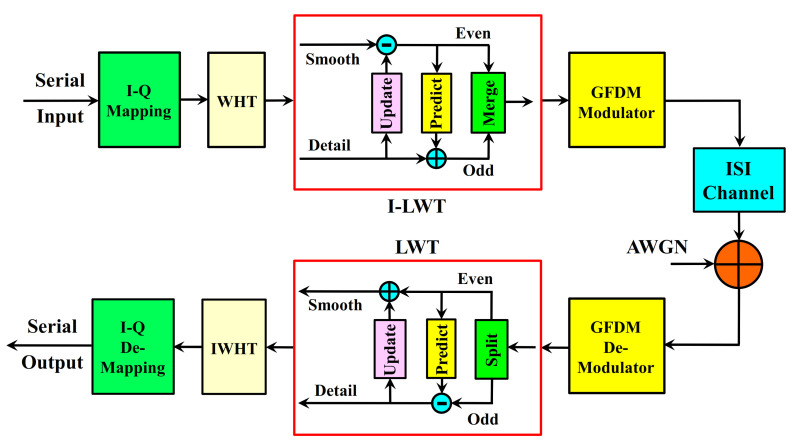
The transceiver structure of the proposed waveform.

- it is a spatial domain method,
- it is easy to use for implementation,
- it allows the use of quicker and in-place computations,
- it allows the use of nonlinear, adaptable, disorderedly sampled and integer to integer wavelet transforms,

Another advantage of the lifting scheme is that the LWT and the reverse-LWT shown in Figure 3 are completely symmetrical to each other. This ensures that LWT can be applied easily.

Filters and subsampling are used in traditional wavelet transform processes. Lifting method was developed by Sweldens in 1996 to reduce the complex mathematical operations used in filtering. Lifting method is the simplest and most efficient method for wavelet transformation [25,26].

In the lifting method, the signal is divided into odd and even samples. Instead of filters, Split Predict and Update processes are applied. Complex calculations are not required for these operations as in traditional methods. In Figure 3, the Lifting wavelet transformation in the LWT unit is given in detail [27].

The original $X\left[k\right]$ signal is primarily separated into odd and even components as in Equations (15) and (16).

(15) $$X_{\mathrm{odd}}\left[k\right]=X\left[2k+1\right]$$

(16) $$X_{\mathrm{even}}\left[k\right]=X\left[2k\right]$$

There is a strong correlation between these two examples. In the predict step, odd samples are tried to be obtained approximately by making use of even samples. The second part, Predict, preserves the high frequency components by eliminating the low frequency components of the signal. In the prediction process, the subset $X_{\mathrm{odd}}\left[k\right]$ is predicted over the subset $X_{\mathrm{even}}\left[k\right]$ using the prediction operator $P\left(.\right)$. The detail information of the $X\left[k\right]$ signal is obtained as in Equation (17) by taking the difference between the subset $X_{\mathrm{odd}}\left[k\right]$ and the predicted $P\left(X_{\mathrm{even}}\left[k\right]\right)$. The $P\left(.\right)$ Prediction operator is a linear combination of a single neighboring subset [26].

(17) $$P\left(X_{\mathrm{even}}\left[k\right]\right)=\underset{i}{\sum }\left(p_{i}X_{\mathrm{even}}\left[k+i\right]\right)$$

Here, $p_{i}$ are the prediction coefficients. This step acts as a high pass filter and the high frequency components ($d\left[k\right]$) that give the detail part of the obtained signal.

(18) $$d\left[k\right]=X_{\mathrm{odd}}\left[k\right]-P\left(X_{\mathrm{even}}\left[k\right]\right)$$

In the update step, the samples are scaled to fix and then added with even samples to provide low pass filtered values for transformation. In the third part, update, the even components are updated by using the detailed signal to reduce the frequency aliasing effect. In the update process, the approximate information about the signal is obtained as a result of collecting the detailed information entering the $U\left(.\right)$ Update operator with the subset $X_{\mathrm{even}}\left[k\right]$. The $U\left(.\right)$ Update operator is a linear combination of neighboring $d\left[k\right]$ values. The formula for the $U\left(.\right)$ Update operator is specified in Equation (19).

(19) $$U\left(d\left[k\right]\right)=\underset{i}{\sum }\left(u_{i}d\left[k+i\right]\right)$$

Here, $u_{i}$ are the updating coefficients. In the update process, the approximate value of the obtained signal by passing the signal through a low pass filter is obtained by updating the linear combination of the $d\left[k\right]$ prediction difference as in Equation (20). These examples contain low frequency components that give approximate information ($s\left[k\right]$) about the signal.

(20) $$s\left[k\right]=X_{\mathrm{even}}\left[k\right]+U\left(d\left[k\right]\right)$$

Inverse transformation is obtained by applying the exact opposite operations used in the conversion to the obtained $d\left[k\right]$ and $s\left[k\right]$ signals. Inverse Lifting wavelet transformation is given in Figure 3 in I-LWT block in detail.

(21) $$X_{\mathrm{odd}}\left[k\right]=d\left[k\right]+P\left(X_{\mathrm{even}}\left[k\right]\right)$$

(22) $$X_{\mathrm{even}}\left[k\right]=s\left[k\right]-U\left(d\left[k\right]\right)$$

After the inverse transformation process, as given in Equation (23), the original signal is obtained by combining the odd index and even index samples with the MRG combination operator.

(23) $$X\left[k\right]=MRG\left\{X_{\mathrm{odd}}\left[k\right],\;X_{\mathrm{even}}\left[k\right]\right\}$$

In addition, Prediction and Update operations are shown in Equation (24) with a 2×2 matrix by performing intermediate operations. Smooth and detail information is obtained from this matrix.

(24) $$\left[\begin{array}{c} d\left[k\right]\\ s\left[k\right] \end{array}\right]=\left[\begin{array}{c} 1\;\;-P\left(.\right)\;\\ U\left(.\right)\;1-P\left(.\right)U\left(.\right) \end{array}\right]\left[\begin{array}{c} X_{\mathrm{odd}}\left[k\right]\\ X_{\mathrm{even}}\left[k\right] \end{array}\right]$$

(25) $$A=\left[\begin{array}{c} 1\;\;-P\left(.\right)\;\\ U\left(.\right)\;1-P\left(.\right)U\left(.\right) \end{array}\right]$$

Since the determinant of the matrix shown by Equation (25) is equal to one, this matrix is an invertible matrix. Since it is the inverse of the matrix, a completely symmetric analysis can be made. The inverse-LWT shown in Figure 3 and indicated by Equations (21) and (22) is mathematically expressed by Equation (26) with a 2×2 matrix. The matrix in Equation (26) is the inverse of the matrix in Equation (24) (shown in Equation (27)).

(26) $$\left[\begin{array}{c} X_{\mathrm{odd}}\left[k\right]\\ X_{\mathrm{even}}\left[k\right] \end{array}\right]=\left[\begin{array}{c} 1-P\left(.\right)U\left(.\right)\;\;P\left(.\right)\\ -U\left(.\right)\;\;\;\;1 \end{array}\right]\left[\begin{array}{c} d\left[k\right]\\ s\left[k\right] \end{array}\right]$$

(27) $$AA=A^{-1}=\left[\begin{array}{c} 1-P\left(.\right)U\left(.\right)\;\;P\left(.\right)\\ \;-U\left(.\right)\;\;\;1 \end{array}\right]$$

To recover the signal, it is sufficient to reverse the operations in Figure 3 in LWT block and change the signs as shown in Figure 3 in I-LWT block.

## 5. The Proposed WHT-LWT GFDM Waveform

The transmitter and the receiver block scheme of the wireless telecommunication scheme using the offered WHT-LWT-GFDM waveform is illustrated in Figure 3 [27]. In this study, the WHT-LWT-GFDM waveform is offered as alternative waveform to OFDM due to the conveniences of LWT and the benefits of WHT, for the 5G and beyond waveform research studies.

In the transmitting region of the WHT-LWT-GFDM scheme depicted in Figure 3, after randomly produced serial input bits are matched via the I-Q mapping unit, they are spread in the WHT unit with the help of Equation (7). In the ILWT block, Inverse Lifting Wavelet Transform is applied to the spread data with the help of Equation (28).

The Inverse Lifting Wavelet Transform of a sequence {*Y(z)*}, can be given by Equation (28).

(28) $$y_{ILWT}\left[n\right]=\overset{Q-1}{\underset{q=0}{\sum }}\overset{Z-1}{\underset{z=0}{\sum }}Y{\left(z\right)}^{q}2^{\frac{q}{2}}\psi \left(2^{q}n-z\right)$$

Here, n is the number of sub-carriers $\left(0\leq n\leq K–1\right)$, $Y\left(z\right)$ demonstrates the data in the wavelet domain and $\psi \left(.\right)$ denotes the mother wavelets with scaling factor q and shift z for each subcarrier.

Inverted LWT transformed bits are applied in GFDM modulator unit to produce WHT-LWT-GFDM data streams. After the acquired WHT-LWT-GFDM data streams are transmitted through the ISI channel and corrupted with AWGN, they arrive to the receiver. Then the deformed data are reconstructed with the proper channel equalization schemes, the recovered data are applied to the GFDM De-Modulator unit. At the output of the GFDM-De-Modulator block, the obtained data are subjected to LWT operation with the assistance of Equation (29).

The Lifting Wavelet Transform of a sequence of $\left\{y\left[n\right]\right\}$, can be given by Equation (29).

(29) $$Y_{LWT}^{q}\left(z\right)=\overset{K-1}{\underset{n=0}{\sum }}y\left[n\right]2^{\frac{z}{2}}\psi \left[2^{z}n-q\right],\;z=0,1,2,\ldots ,K-1,\;q=0,1,2,\ldots ,K-1$$

Here, $y\left[n\right]$ denotes the data in the time domain.

After the data arrives at the output of the LWT unit, the data spread to sub-carriers is combined with the help of Equation (8) in the IWHT unit. After the acquired data at the output of the IWHT block are de-modulated in the reverse I-Q mapping unit, the desired benchmark metric, as for example the bit-error rate (BER) or the SER, are computed by trying to acquire the information sent by the transmitter.

**Figure 4:**
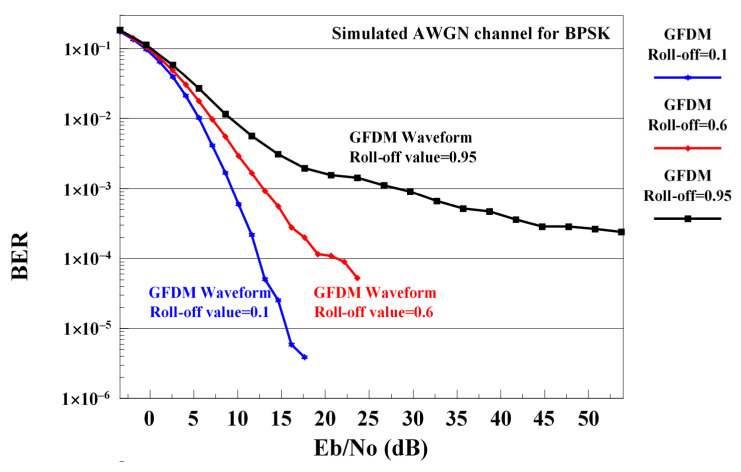
The effects of different roll-off values on the BER performance of GFDM waveform in AWGN channel.

The choice of both K and M even is known to be problematic in GFDM, this choice along with a high roll-off is even worst [28,29,30]. When a new GFDM-based system is to be designed for the future, it is necessary to correct the negative effect of high roll-off values on GFDM to make accurate analyzes according to different roll-off values within the existing conditions. This is one of the aims of this study. Therefore, it is very important to increase the performance of the GFDM waveform at high roll-off values. The main contribution of this study is to solve this issue with the use of LWT. The effects of different roll-off values in the RC pulse shaping filter on the BER performance of the GFDM waveform are shown in Figure 4.

When Figure 4 is examined, it is seen that the performance deteriorates as the roll-off value of the RC pulse shaping filter used in the GFDM waveform increases. Increasing the performance of GFDM and WHT-GFDM waveforms with the proposed method at high roll-off values is the biggest contribution of this study.

### Computational Complexity Analysis

Computational complexity must be known at each iteration time to examine the advantages and disadvantages of different communication systems against each other. In this study, computational complexity is defined as the number of multiplications and additions taken to process the data. Each number of additions and multiplications are calculated separately.

In the proposed study, WHT is used. To calculate the WHT conversion, a total of $\left(K\log_{2}K\right)$ real multiplication operations on the transmitter side and $\left(K\log_{2}K\right)+K$ real multiplication operations are required on the receiver side. Here, K is the sub-carriers. To calculate the LWT transformation, a total of $K\left(P+U\right)/2$ real multiplications and $K\left(P+U\right)/2$ real addition operations on the transmitter side and $K\left(P+U\right)/2$ real multiplications and $K\left(P+U\right)/2$ real addition operations are required on the receiver side. Here, P is the length of prediction coefficients sequence given by Equation (17) and U is the length of update coefficients sequence given by Equation (19). Therefore, the computational complexity of the proposed method varies according to the length of the filter coefficients (mother wavelets such as Haar, lazy and bior) used in the Update and Predict stages. If these calculations are adapted to the GFDM waveform, the computational complexity of the proposed method can be obtained as in Table 1. In Table 1, M shows the sub-symbols and L indicates the span of receiver filter in the neighborhood of each subcarrier band [8]. The number of addition operations are about the same number of multiplication operations.

## 6. Simulation Results and Discussions

The computer simulations section contains three parts. In the first part, the traditional OFDM, GFDM, WHT-GFDM [18] and the proposed WHT-LWT-GFDM method are compared in the AWGN channel environment. In the second part, comparative performance analysis of four methods is performed in the frequency flat Rayleigh fading channel environment. PAPR improvement of the above schemes are evaluated in the third part of the section. Computer simulations are performed by independent 100 Monte Carlo cycles for Binary Phase Shift Keying (BPSK) modulation. In all simulations, the roll-off factor of the RC pulse shaping filter was 0.95, and the simulations are carried out using K=64 sub-carriers and M=8 sub-symbols for GFDM and the proposed waveform. BER metric is employed in the first and the second part of simulations.

In this study, OFDM waveform is created according to the IEEE 802.11a/HIPERLAN/2 standard. Accordingly, 48 sub-carriers carry data in an OFDM symbol, while 4 sub-carriers are used as pilots. 12 sub-carriers are also sent as empty. Accordingly, after creating 64 complex samples, time domain samples are obtained with the help of IFFT. In the classical OFDM system, the last 16 of the time domain samples are copied and added to the head to prevent ISI. By adding these 16 samples, called CP, an OFDM symbol consisting of a total of 80 samples is obtained. 100 realizations of this OFDM signal is generated, resulting in a total length of 100 × 80 = 8000 bits OFDM data packets.

For the GFDM waveform, as described in detail in Section 2, the roll-off factor of the RC pulse shaping filter is 0.95 and the GFDM symbol consisting of 512 samples and M=8 sub-symbols and K=64 sub-carriers are obtained. A total of 20 × 512 = 10,240 length GFDM data packets are obtained by generating 20 of this GFDM signal.

WHT-GFDM and WHT-LWT-GFDM waveforms are also produced in the same way. In the WHT-GFDM waveform, data that are modulated differently from GFDM are subjected to WHT transform, and data packets of the same length are formed. The proposed WHT-LWT-GFDM data packets are also subjected to I-LWT transformation after WHT conversion in the WHT-GFDM waveform, and then data packets of the same length are formed.

CP was not used in GFDM waveform, WHT-GFDM waveform, and the recommended waveform.

### 6.1. AWGN Channel Simulation Results

**Figure 5:**
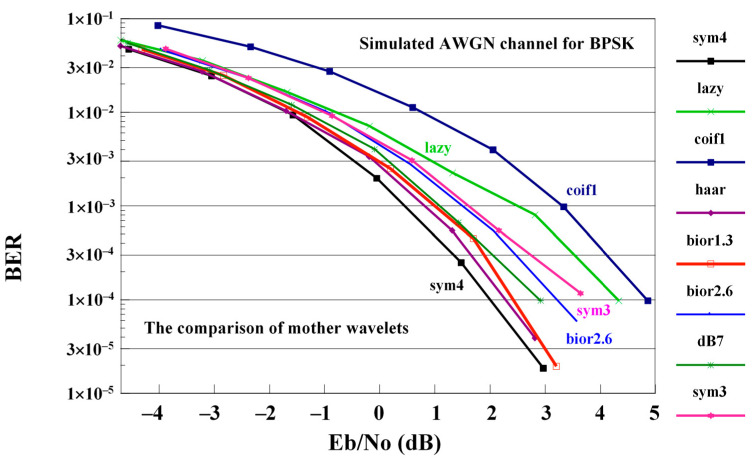
The comparison of performance of the proposed WHT-LWT-GFDM obtained using different mother wavelets on the AWGN channel.

The outcomes of traditional OFDM, GFDM, WHT-GFM [18] and the proposed WHT-LWT-GFDM methods are compared on the AWGN channel. The influences of various wavelet families of the varied wavelet families, for example bior1.3, bior2.6, coif1, Haar, db7, sym3, lazy and sym4, are examined on the proposed technique. The performance of the proposed waveform with various mother wavelets is shown in Figure 5.

Figure 5 shows performance comparison whose conclusion is that the best mother wavelets in case of AWGN channel is sym4 and the worst mother wavelets in this case is the coif1 mother wavelet in this case. The performance of the proposed waveform in the case of the other mother wavelets is between the performance of the proposed waveform obtained using sym4 and coif1 mother wavelets.

**Figure 6:**
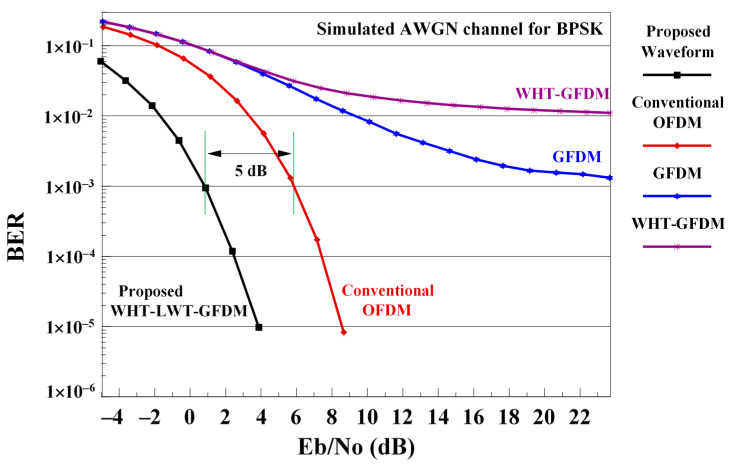
Comparison of traditional OFDM, GFDM, WHT-LWT-GFDM, and proposed WHT-LWT-GFDM waveforms performance on AWGN channel.

In Figure 6, the performance of the conventional OFDM, GFDM, WHT-GFDM [18] and the proposed WHT-LWT-GFDM using sym4 mother wavelets are given in the AWGN channel as BER-SNR (Signal to Noise Radio) curve comparisons for 1000 Monte Carlo simulations.

Figure 6 illustrates the BER-SNR curves of traditional OFDM, GFDM, WHT-GFDM [18] and the proposed method in the AWGN channel. Analyzing the figure, it can be observed that the GFDM waveform converges to nearly 1 × 10^−3^ error floor. Although the WHT-GFDM method achieves the same performance as the GFDM up to roughly 6 dB of SNR value, it converges to the base of the 1 × 10^−2^ error floor after 6 dB of SNR value. The classical OFDM system overcomes the GFDM system and eliminates the error floor. On the other hand, the proposed waveform eliminates the error floor by outperforming both the classical OFDM, WHT-GFDM [18] and GFDM waveform, and provides an approximately 5 dB of SNR gain versus the traditional OFDM for the 1 × 10^−3^ BER worth. This enhancement is obtained as a consequence of the application of both Walsh–Hadamard transform and lifting wavelet transformation. Additionally, this improvement can be explained by using twice as much data in the proposed system with zero padding components compared to the OFDM system. This also reduces the effect of errors caused by noise and disturbing effects of the channel by spreading the sent data. It can also be explained by the fact that this improvement meets the excellent reconstruction feature since the wavelets have orthonormal structure.

### 6.2. Flat Rayleigh Fading Channel Simulation Results

**Figure 7:**
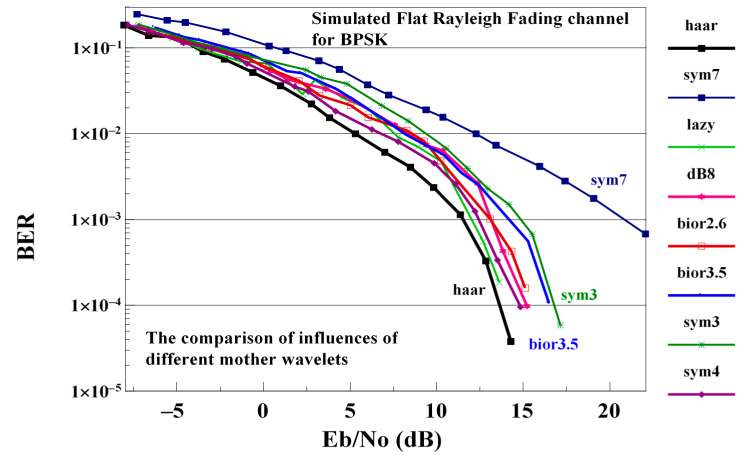
A comparison of the performance of the proposed waveform in case of flat Rayleigh fading channel obtained using different mother wavelets.

In the following, the performance of classical OFDM, GFDM, WHT-GFDM [18] and WHT-LWT-GFDM methods are compared on flat Rayleigh fading channel. In this work, the influences of varied wavelet families as an example of bior2.6, bior3.5, Haar, db8, sym3, sym4, lazy, and sym7 on the performance of the offered scheme are examined. The performance of the proposed waveform with various mother wavelets is shown in Figure 7.

The best performance of the waveform proposed is obtained using the Haar mother wavelets and the worst performance is obtained using the sym7 mother wavelets. The performance obtained using another mother wavelet is between the performance obtained using Haar or sym7 mother wavelets. Hence, in the rest of this sub-section, the proposed waveform is implemented using the Haar mother wavelets.

**Figure 8:**
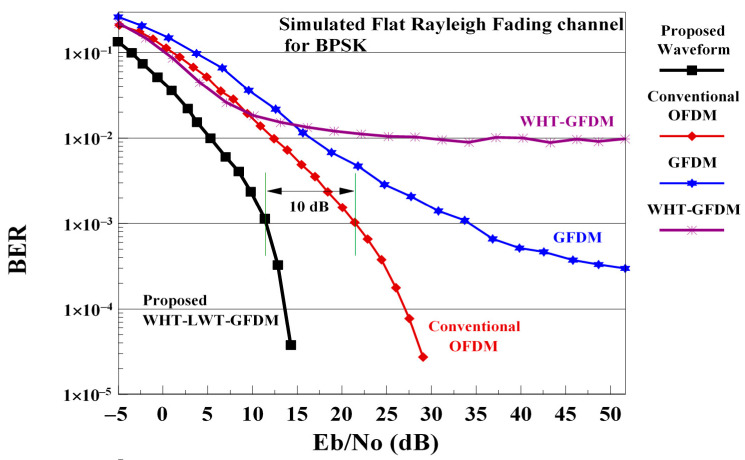
The BER-SNR comparisons of the traditional OFDM, GFDM, WHT-GFDM, and WHT-LWT-GFDM implemented using Haar mother wavelets waveforms on the flat Rayleigh fading channel.

In Figure 8, we compare the performance of classical OFDM, GFDM, WHT-GFDM [18] and proposed waveform, implemented using Haar mother wavelets on flat Rayleigh fading channel, using the BER-SNR metric.

As Figure 8 shows, the BER-SNR curve which corresponds to the GFDM waveform converges slightly below the 1 × 10^−3^ floor after 35 dB of SNR value. Although the WHT-GFDM waveform performs better than GFDM and conventional OFDM at low SNR values, it converges to the floor error of 1 × 10^−2^ after 20 dB. The conventional OFDM waveform is better than GFDM and has not an error floor. However, the proposed waveform overcomes both the classical OFDM, WHT-GFDM [18] and the GFDM waveforms, eliminating the error floor and providing an approximately 10 dB of SNR gain versus the conventional OFDM for the 1 × 10^−3^ BER value. Achieving a roughly 10 dB of SNR enhancement on the frequency flat Rayleigh fading channel with the proposed scheme is extremely satisfactory. Additionally, this improvement can be explained by the fact that LWT has good time-frequency localization features, ICI and ISI suppression, and flexibility. It can also be explained by the fact that this improvement meets the excellent reconstruction feature since the wavelets have orthonormal structure.

### 6.3. PAPR Simulation Results

In this sub-section, the PAPR performance of the waveforms mentioned above are compared. PAPR simulations consist of two parts. In the first part, PAPR simulation studies on the transmitter side, and in the second part, PAPR simulation studies on the receiver side at different SNR values are made. PAPR is defined in the next equation.

(30) $$PAPR_{dB}\left(x\left[n\right]\right)=10\log_{10}\left(\frac{\underset{0\leq n\leq NL-1}{\max}{\left|x\left[n\right]\right|}^{2}}{E\left[{\left|x\left[n\right]\right|}^{2}\right]}\right)$$

Here, $X\left[n\right]$ denotes the sent data of the systems mentioned above, NL is the oversampling factor, $\max\left[.\right]$ denotes the maximum value and the operator $E\left[.\right]$ denotes the statistical mean (expectation). The PAPR performance is evaluated using the Complementary Cumulative Distribution Function (CCDF) of PAPR. Considering the PAPR threshold $PAPR_{0}>0$, the probability $PAPR_{dB}$ that the PAPR is higher than the threshold is computed by CCDF and is expressed in the following equation:

(31) $$CCDF(PAPR_{0})=Pr\left\{PAPR_{dB}>PAPR_{0}\right\}$$

Here, Pr denotes the probability. PAPR simulations were carried out using 1000 realizations of the above-mentioned waveforms. In all PAPR simulation studies, the roll-off factor of the RC pulse shaping filter was 0.1, and was carried out using K=64 sub-carriers and M=8 sub-symbols for GFDM, WHT-GFDM, and the proposed waveforms.

#### 6.3.1. PAPR Simulation Results on Transmitter Side

This section shows the PAPR simulation results on the transmitter side to verify the performance of the proposed waveform and compare with other waveforms.

**Figure 9:**
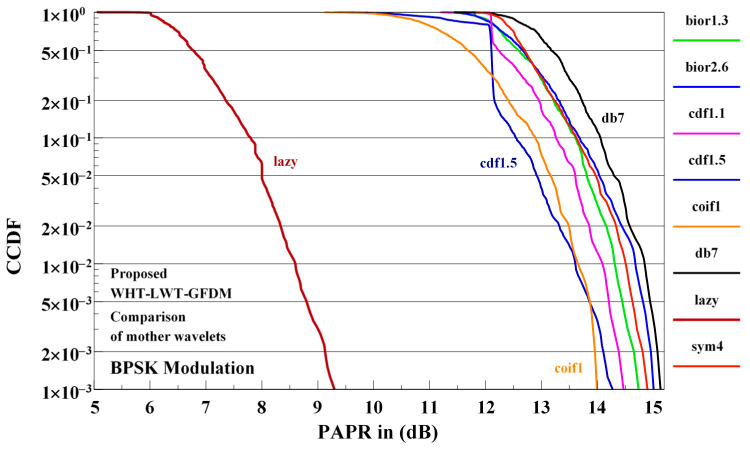
The effects of the different mother wavelets on PAPR performance of proposed WHT-LWT-GFDM waveform for BPSK modulation.

The effects of the various mother wavelets on the PAPR performance of the WHT-LWT-GFDM waveform proposed for BPSK modulation are shown in Figure 9.

In Figure 9, the influences of various mother wavelets as for example bior1.3, bior2.6, cdf1.1, cdf1.5, coif1, db7, lazy, and sym4 on the PAPR performance of the proposed waveform for BPSK modulation are presented. The best performance is obtained using the lazy mother wavelet and the worst performance is obtained using the db7 mother wavelets. The PAPR performance of the proposed waveform when one of the other mother wavelets considered is used falls between the performance obtained using the lazy or the db7 mother wavelets. Since the best PAPR performance of the proposed waveform is obtained using the lazy mother wavelets, we will use in the rest of this sub-section this mother wavelets for the implementation of the proposed waveform.

**Figure 10:**
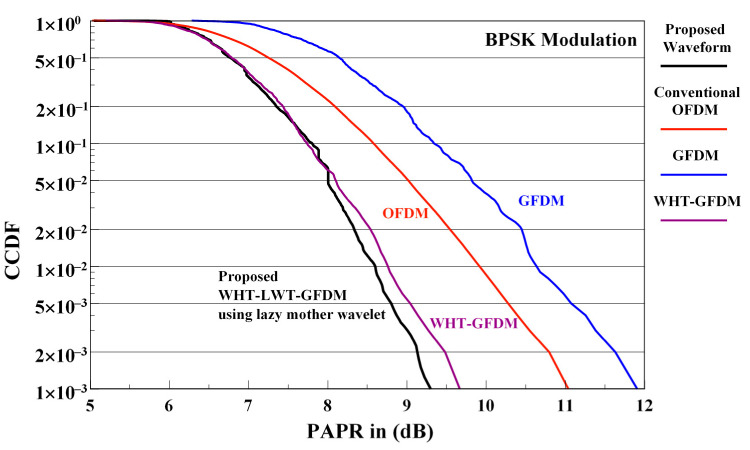
The PAPR performance comparison of the conventional OFDM, GFDM, WHT-GFDM, and WHT-LWT-GFDM waveforms.

The comparison of PAPR performance of the conventional OFDM, GFDM, WHT-GFDM, and proposed waveforms is illustrated in Figure 10 for the BPSK modulation.

When the PAPR performance given in Figure 10 are examined, it is noticed that the WHT-GFDM waveform surpasses conventional OFDM and GFDM waveforms. For 1 × 10^−3^ CCDF value, it is seen that it gives approximately 1.3 dB PAPR enhancement versus the conventional OFDM waveform and approximately 2.3 dB PAPR enhancement versus the GFDM waveform. However, for 1 × 10^−3^ CCDF value, it is observed that the proposed WHT-LWT-GFDM waveform provides approximately 0.4 dB PAPR gain versus WHT-GFDM waveform, provides roughly 1.5 dB PAPR enhancement versus conventional OFDM waveform and gives approximately 2.5 dB PAPR enhancement versus GFDM waveform. On the other hand, there appears to be a trade-off in the WHT-GFDM waveform. Although unsuccessful results were acquired in BER-SNR curve with the WHT-GFDM waveform, good results are obtained for PAPR.

#### 6.3.2. PAPR Simulation Results on Receiver Side

We now investigate the effect of channel noise on the PAPR performance of the waveforms considered in this paper. PAPR performance of each of the four waveforms considered are compared for 4-QAM and 16-QAM modulation at 0 dB, 5 dB, 10 dB, 15 dB, 20 dB, and 25 dB SNR values.

**Figure 11:**
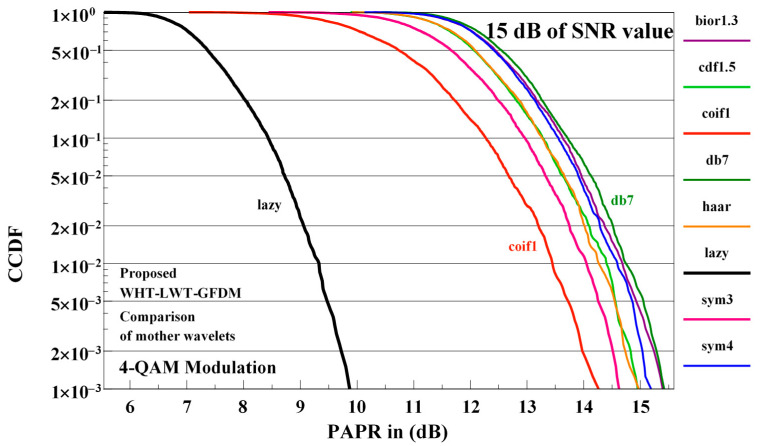
The influences of the different mother wavelets on PAPR performance of proposed waveform in 15 dB of SNR value for 4-QAM signal.

The effects of different mother wavelets on the PAPR performance of the proposed waveform are shown in Figure 11 for 4-QAM modulation at 15 dB SNR value on the receiver side.

The best PAPR performance is obtained when the proposed waveform is implemented using the lazy wavelet and the worst PAPR performance is obtained using the db7 mother wavelets. The performance of the proposed waveform implemented using the other mother wavelets considered is between the performance obtained using these two mother wavelets. Because the best performance of the proposed waveform is obtained using the lazy mother wavelets, in the rest of this sub-section we will use this mother wavelets.

**Figure 12:**
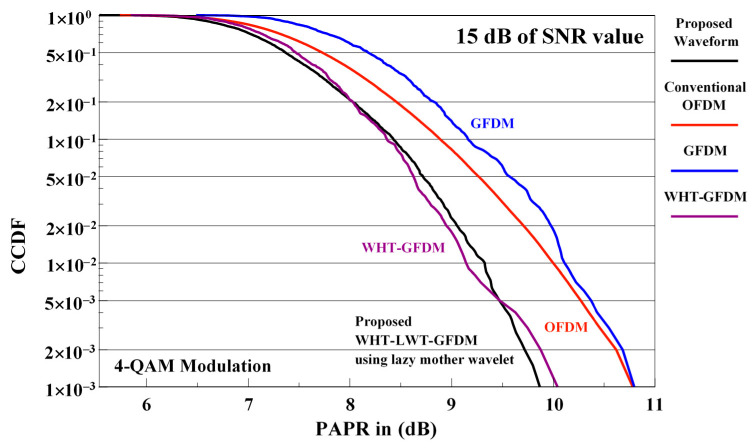
The PAPR performance comparisons of conventional OFDM, GFDM, WHT-GFDM, and WHT-LWT-GFDM waveforms in case of 4-QAM modulation for a SNR of 15 dB at receiver side.

The comparison of PAPR performance curves at receive side for conventional OFDM, GFDM, WHT-GFDM, and proposed waveforms is shown in Figure 12 for 4-QAM modulation and SNR of 15 dB.

From Figure 12, we can observe that the proposed waveform outperforms the conventional OFDM and GFDM waveforms, and gives approximately 1.0 dB PAPR gain for 1 × 10^−3^ CCDF level. On the other hand, it is understood that the proposed waveform and the WHT-GFDM waveform exhibit approximately the same performance.

**Figure 13:**
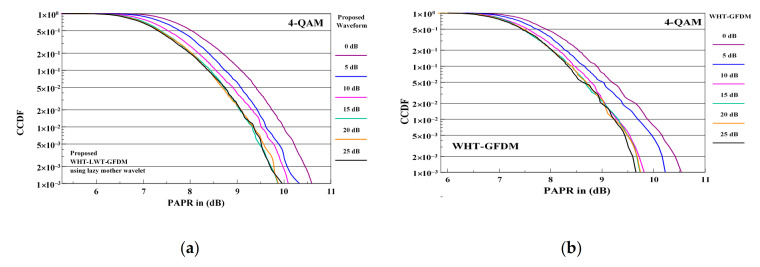
The PAPR performance comparison of the proposed waveform (**a**) and WHT-GFDM waveform (**b**) in between 0–25 dB of SNR values for 4-QAM modulation.

The comparison of PAPR performance curves of the proposed waveform and WHT-GFDM waveform is shown in Figure 13 for 4-QAM modulation and SNR values at receiver side between 0 and 25 dB.

When PAPR performances in Figure 13 are examined, it is seen that PAPR dB gain is provided from 0 dB to 15 dB of SNR values with both waveforms. However, it is understood that PAPR dB gains are approximately the same after 15 dB of SNR value. In other words, it is observed that no PAPR dB gain is provided after 15 dB of SNR value.

**Figure 14:**
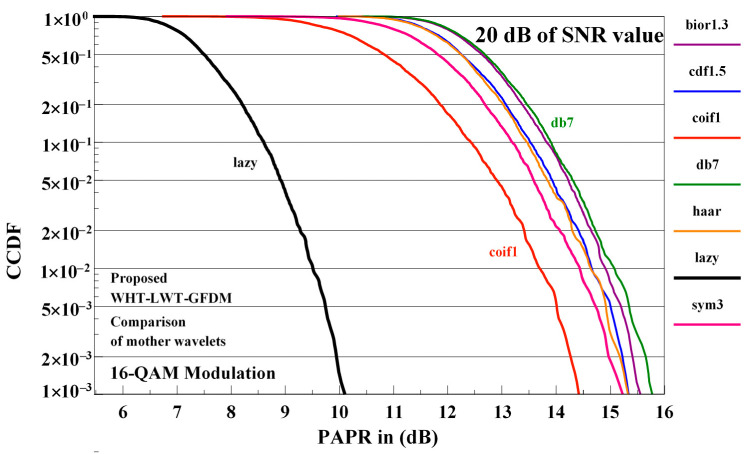
The influences of the different mother wavelets on PAPR performance of proposed waveform in 20 dB of SNR value for 16-QAM modulation.

The influences of diverse mother wavelets on the PAPR performance of the proposed waveform are shown in Figure 14 for 16-QAM modulation at 20 dB SNR value on the receiver side.

Investigating Figure 14, it is observed that the best outcome is obtained using the lazy mother wavelets and the worst outcome is obtained using the db7 mother wavelets. The PAPR performance of the proposed waveform at the receiver side obtained using the other mother wavelets considered is between the performance obtained using these two mother wavelets. As the best performance of the proposed waveform is obtained using the lazy mother wavelets, it will be used in the following to compare the proposed waveform with other waveforms.

**Figure 15:**
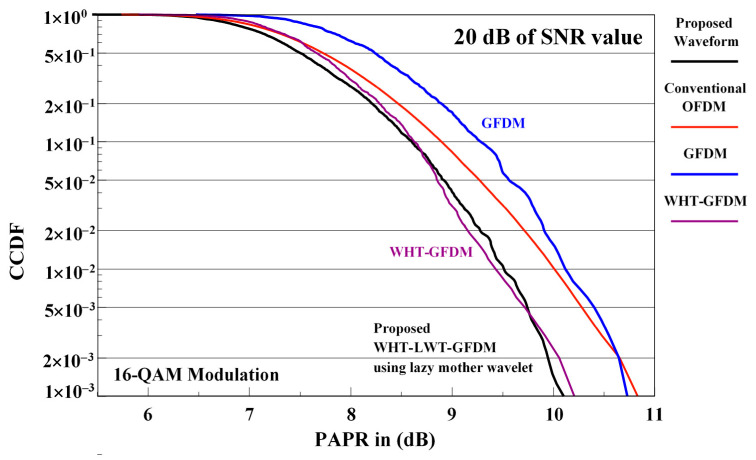
The PAPR performance comparisons of the conventional OFDM, GFDM, WHT-GFDM, and WHT-LWT-GFDM waveforms for a SNR value of 20 dB for 16-QAM modulation.

The comparison of PAPR performance curves of conventional OFDM, GFDM and WHT-GFDM waveforms with the proposed waveform is given in Figure 15 for 16-QAM modulation at 20 dB SNR value in the receiver.

Analyzing Figure 15, it is observed that the proposed waveform outperforms the conventional OFDM and GFDM waveforms and gives nearly 0.6 dB PAPR enhancement for 1 × 10^−3^ CCDF level. On the other hand, it is understood that the proposed waveform and the WHT-GFDM waveform perform very close to each other.

**Figure 16:**
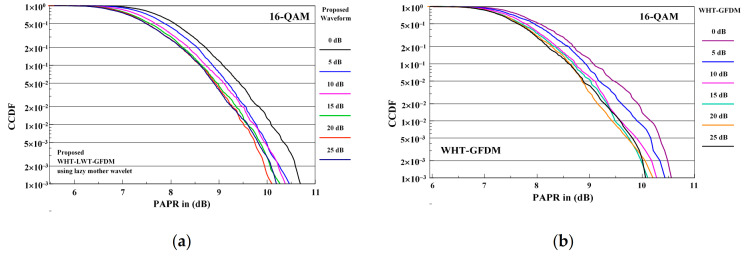
The PAPR performance comparison of the proposed waveform (**a**) and WHT-GFDM waveform (**b**) in between 0–25 dB of SNR values for 16-QAM modulation.

The comparison of PAPR performance curves of the proposed waveform and WHT-GFDM waveform is given in Figure 16 for 16-QAM modulation considering the interval of SNR values at the receiver side between 0 and 25 dB.

When PAPR performances in Figure 16 are examined, it is seen that similar to 4-QAM modulation, PAPR dB gain from 0 dB to 15 dB of SNR values is provided with both waveforms. However, it is understood that the PAPR dB gains are approximately the same after 15 dB SNR value. In other words, it is observed that there is no PAPR dB gain after 15 dB SNR value.

## 7. Conclusions

In this study, a novel cascaded Walsh–Hadamard Transform and Lifting Wavelet Transform-based GFDM waveform design is proposed as an alternative to the OFDM for 5G and beyond. The proposed waveform is obtained as a union of WHT and LWT with GFDM waveform. Computer simulations are carried out to evaluate the performance of the proposed waveform and to realize comparisons with conventional OFDM, GFDM, and WHT-GFDM waveforms. The gain in performance obtained using the proposed waveform is substantial. The proposed waveform has a SNR gain of 10 dB on flat Rayleigh fading channel and a SNR gain of 5 dB on AWGN channel. By using the WHT-LWT-GFDM, the proposed waveform has the additional benefit of improving PAPR performance. Without compromising BER performance, satisfactory gains in PAPR performances have also been obtained with the proposed waveform. Moreover, this waveform can be generated by systems with controlled complexity and it can be easily applied in the next generation of wireless telecommunication systems.

## Figures and Tables

**Table 1:** Computational complexities of the waveform for AWGN channel.

Methods	Multiplication
OFDM Transmitter	$\left(K\log_{2}K-3K+4\right)$
OFDM Receiver	$\left(K\log_{2}K-3K+4\right)$
GFDM Transmitter [8]	${\left(KM\right)}^{2}$
Matched Filter GFDM Receiver [8]	$KM\left(\log_{2}KM+\log_{2}M+L\right)$
WHT-GFDM Transmitter	${\left(KM\right)}^{2}+KM\log_{2}KM$
Matched Filter WHT-GFDM Receiver	$KM\left(\log_{2}KM+\log_{2}M+L\right)+KM\log_{2}KM+KM$
WHT-LWT-GFDM Transmitter	${\left(KM\right)}^{2}+KM\log_{2}KM+KM\left(P+U\right)/2$
Matched Filter WHT-LWT-GFDM Receiver	$KM\left(\log_{2}KM+\log_{2}M+L\right)+KM\log_{2}KM+KM+KM\left(P+U\right)/2$

## Data Availability

Not applicable.
